# Evaluation of Segmentation, Rotation, and Geographic Delivery Approaches for Deployment of Multiple First-Line Treatment (MFT) to Respond to Antimalarial Drug Resistance in Africa: A Qualitative Study in Seven Sub-Sahara Countries

**DOI:** 10.3390/tropicalmed9050093

**Published:** 2024-04-23

**Authors:** Celine Audibert, Adam Aspinall, Andre-Marie Tchouatieu, Pierre Hugo

**Affiliations:** Medicines for Malaria Venture (MMV), Route de Pre-Bois 20, 1215 Meyrin, Switzerland; aspinalla@mmv.org (A.A.);

**Keywords:** uncomplicated malaria, malaria drug resistance, multiple first-line treatment, sub-Saharan Africa, qualitative survey

## Abstract

Background: Several studies recently confirmed the emergence of resistance to antimalarial drugs in sub-Saharan Africa. Multiple first-line treatment (MFT) is one of the measures envisaged to respond to the emergence and spread of this resistance. The aim of this study was to identify the perceived advantages and disadvantages of several MFT deployment strategies and to better understand potential implementation drivers and barriers. Methods: A qualitative survey was conducted in seven sub-Saharan countries amongst key opinion leaders, national decision makers, and end users. A total of 200 individual interviews were conducted and findings were analyzed following a thematic inductive approach. Results: From a policy perspective, the new MFT intervention would require endorsement at the global, national, and regional levels to ensure its inclusion in guidelines. Funding of the MFT intervention could be a bottleneck due to costs associated with additional training of healthcare workers, adaptation of drug delivery mechanisms, and higher costs of drugs. Concerning the MFT deployment strategies, a slight preference for the segmentation strategy was expressed over the rotation and geographic approaches, due to the perception that a segmentation approach is already in place at country level. Conclusions: The findings highlighted the need for a collective approach to MFT deployment through the engagement of stakeholders at all levels of malaria management.

## 1. Introduction

Malaria remains a major public health issue globally with an estimated 249 million cases and 608,000 deaths reported in 2022 [1]. Most of these cases and deaths are seen in sub-Saharan Africa (SSA), with nearly 80% of all malaria deaths occurring among children under the age of 5 [1]). Since 2005, artemisinin-based combination therapies (ACTs) have been recommended as the first-line treatment for uncomplicated malaria caused by *Plasmodium falciparum* because of their well-tolerated and rapid-efficacy profile [2,3]. Adoption of ACTs was prompted by malaria treatment failure caused by the worldwide spread of malaria parasite resistance to chloroquine (CQ) and sulfadoxine-pyrimethamine (SP) [4,5]. Several studies recently confirmed the emergence of artemisinin resistance mutations in SSA countries, which increases the pressure on the partner drug and could result in treatment failure [6,7,8,9,10]. These concerns prompted the WHO to recently publish a new resistance mitigation strategy calling for countries to adopt proactive measures such as diversification of ACTs [11].

One approach to diversification of ACT use is the deployment of multiple first-line treatments (MFT). For over 15 years, MFT modelling has suggested that it can contribute meaningfully to slow the emergence and spread of antimalarial drug resistance in Africa [12]. In MFT approaches, at least two ACTs are simultaneously deployed in first-line treatment. This strategy may delay the emergence of resistance by decreasing the pressure on the partner drugs [13,14]. Importantly, the ability of MFT to prolong the lifespan of existing ACTs has only been demonstrated using mathematical transmission models [13,15,16], because designing field trials to measure an outcome that may take decades to occur would be very complex and expensive [13]. Similarly, various MFT deployment strategies have been envisaged, but none of them have been tested at scale yet. These strategies include (a) rotation during which an ACT is scheduled to be used for a certain duration of time before being replaced by another one, or until a certain resistance threshold is observed; (b) segmentation, where different ACTs are given to different segments of the population (pediatric, pregnant women, adults), or through different distribution channels (clinics, pharmacies, health centers); (c) geographic, where different ACTs are distributed to different administrative regions [13,17].

Each MFT deployment strategy has its own specificities, advantages, and constraints. Embedding them into existing healthcare systems presents challenges in terms of distribution, planning, logistics management, and stakeholder acceptability, and requires the creation of policies that can facilitate the adoption of the intervention [13]. Feasibility and acceptability studies can help identify potential barriers and facilitators of implementation. A recent pilot conducted in Burkina Faso investigated stakeholders’ perceptions of an MFT intervention that relied on a segmentation approach [18]. Authors found that the adherence by both the population and key stakeholders would depend on treatment efficacy, low severity level of side effects, cost, and drug availability. In addition, they also showed that such an intervention was operationally feasible and acceptable by stakeholders in the health system in Burkina Faso [17].

To further explore African countries’ readiness to adopt MFT, a qualitative survey was conducted in seven SSA malaria endemic countries. Importantly, the survey assessed the acceptability of various MFT deployment strategies from the point of view of those who provide direct care to patients, here referred to as end users. The study aims were to identify perceived advantages and disadvantages of each strategy and to better understand the drivers and barriers for potential implementation. Ultimately, the findings from this survey may help inform policy makers on which deployment strategy is most appropriate in their specific context.

## 2. Methods

### 2.1. Study Setting

The study was conducted in seven SSA countries. Countries were selected based on their mix of geographical location, malaria burden, and number of ACTs registered for first-line treatment. Table 1 shows the characteristics of the selected countries. Malaria burden was determined based on WHO’s identification of countries with the highest malaria burden and included in the High Burden to High Impact initiative [19]. ACT registrations for first-line treatment reflect the status at the time of the study for each country.

Countries conducting MFT pilots at the time of the study (Kenya and Burkina Faso) were excluded because stakeholders’ perceptions and attitudes towards MFT had already been investigated and published [18,20]. The perceptions of MFT in countries that did not have practical experience with the intervention were prioritized, to identify potential barriers and drivers of implementation in advance.

### 2.2. Research Design

The study used a qualitative approach to data collection, involving in-depth, one-on-one interviews with national stakeholders engaged in malaria control and elimination in the fight against malaria, and end users. This approach was selected given the exploratory nature of the topics to be investigated. The study consisted of semi-structured discussion guides that were adapted to the type of respondent [provided in Supplementary Materials]. The discussion guides investigated current awareness and perceptions of resistance to ACTs prior to investigating three MFT scenarios—segmentation, rotation, and geographic approaches. A short description of these three scenarios was provided to each participant to ensure that they all had a similar basic understanding of MFT. A description of each approach is shown in Box 1. The perceived advantages and disadvantages of each approach were investigated, and respondents were asked to select their preferred scenario and to explain their choice. Potential challenges to implementation were also investigated.

Box 1 **Description of the three MFT delivery approaches as presented at the time of the study.**

Segmentation approach

A trial in Kaya district, Burkina Faso, using the segmentation approach recruited its first patients in December 2019. The trial is designed as follows: patients seeking care at the community level receive artemether-lumefantrine (AL) regardless of age, gender, or pregnancy status, patients seeking care at the health facility level receive pyronaridine-artesunate (PyAS) if they are under 5 years of age, while pregnant women receive AL and all other patients receive dihydroartemisin–piperaquine (DHA-PQ).

Rotation approach

Kenya has set up an alternative (rotational) approach in which one county rotates AL, artesunate–amodiaquine (AS-AQ) and DHA-PQ sequentially for 8 months each, (8 months of AL, followed by 8 months of AS-AQ, followed by 8 months of DHA-PQ), a second county rotates AL for 12 months followed by pyronaridine artesunate (PyAS) for 12 months, while a third county serves as a control using AL for the full duration of the trial (24 months).

Geographic approach

The distribution of different drugs in different geographical regions.

### 2.3. Respondent Selection, Sample Size and Composition

Respondents across the malaria management environment were identified and invited to participate in the survey. The key informants were divided into two overarching categories to facilitate the analysis: (1) central-level participants, consisting of National Malaria Control Program (NMCP) or Ministry of Health (MoH) representatives, academic researchers, and development partners, and (2) end users, consisting of physicians, pharmacists, nurses, and community health workers (CHWs) who are in charge with delivering care to patients. The rationale for this categorization was that each group represented specific parts of the malaria management landscape. Central-level participants are best positioned to reflect on policy procedures and the regulatory context for the uptake of a new intervention, whereas end users provide the most relevant insights on the practical implementation of the intervention. Central-level participants were recruited through a mix of purposive and snowball methods. End users were recruited through convenience sampling from districts and health facilities with a medium to high malaria burden. Potential participants were approached either by phone or email. Screening questions were used to qualify participants and ensure that they had sufficient knowledge or involvement with malaria management. Recruitment was stopped when the saturation point was reached, that is when no new information was obtained. A total of 32 central-level participants and 168 end users were interviewed. Table 2 provides a breakdown of the participants per country and per respondent category.

### 2.4. Data Collection

Two semi-structured discussion guides were prepared to facilitate data collection. Preparatory meetings were held among members of the research team and the field partners prior to data collection, and each interviewer received full training on the study material. The guides were piloted with a sub-set of respondents and were adapted to improve the interview flow and facilitate the collection of information. The research team and the interviewers had no established relationship with study participants. Interviews were carried out either face to face or remotely via telephone, Zoom, Skype, or WhatsApp depending on the participant’s preference. All interviews were carried out in the participant’s native language. Each interview lasted between 20 and 60 min and were audio-recorded to facilitate translation, transcription, and analysis. Interviews took place from September 2020 to March 2021.

### 2.5. Data Analysis

The tape recordings were transcribed, and all non-English transcripts were translated to English for analysis. The transcripts were uploaded to NVivo 10 (QSR International Pty Ltd., Burlington, MA, USA) software and subjected to a process of coding. The coding process followed the three-step thematic inductive approach described by Thomas and Harden (2008) [21] consisting of the following: (1) coding of text, (2) development of descriptive themes, and (3) generation of analytical themes. For each target group, two researchers coded the data independently and compared the output for agreement and construction of a codebook. In a second round of coding, all themes were merged into overarching categories, the reflections were extracted per respondent group, and storylines were written for each target group.

### 2.6. Ethical Approval

The protocol and data collection instruments were reviewed and approved by the Comité National d’Ethique pour la Recherche en Santé (CNERS), approval code 00 000 233 MSAS/CNERS/Sec, protocol SEN20/72 and approval date 7 December 2020. Separate local ethics clearance was obtained for each country by the field partners. The objective of the study was described before each interview and written informed consent was sought from each participant. Confidentiality was assured at all stages of the study and permission was asked for tape-recording. The study did not involve patients and did not collect patient characteristics. As such, there was no institutional review board involved in approving the research.

## 3. Results

### 3.1. Awareness and Perception of Resistance to ACTs

Prior to investigating participants’ responses to the different MFT delivery approaches, the survey explored their awareness and perception of resistance to ACTs. At the time of the study, most central-level participants believed that there was no significant resistance to ACTs in their country. This perception was based on regular therapeutic efficacy studies (TES) that demonstrated good sensitivity of the parasite to available ACTs at the time of the study. Others indicate the absence of convincing evidence showing malaria resistance to ACTs, especially for respondents from DRC and Côte d’Ivoire. Central-level participants from Uganda, Tanzania, and Nigeria flagged the absence of up-to-date data about malaria resistance, leading to their inability to estimate the level of resistance to ACTs. Despite the lack of strong evidence on resistance to ACTs, central-level participants were concerned by the threat that *P. falciparum* resistance to artemisinin would cause.

*“We also recognize the fact that this [resistance] is happening in some parts of the globe, in Asia. I also know that Rwanda is also registering some kind of resistance to ACTs”.*

(NMCP, Nigeria)

If resistance were to emerge, central-level participants feared that it would erode the gains made in malaria elimination and lead to the loss of one of the most effective weapons against the disease. According to central-level participants, potential drivers of artemisinin resistance include poor treatment practices with healthcare providers treating any fever as malaria (Cameroon), some clinicians still prescribing chloroquine suggesting poor compliance to treatment guidelines (Nigeria), and weak regulation of the private sector despite its significant role in malaria treatment (DRC).

From the end users’ point of views, while most of the participants believed that resistance to ACTs was minimal in their practice, they regularly experienced treatment failures. The treatment failures were largely attributed to other causes than antimalarial drug resistance, and included a broad range of causes, such as self-medication, wrong dosage, counterfeit products, and poor compliance.

*“They say there is resistance, but it’s just lack of maintenance of the protocol”.*

(CHW, Mali)

*“I’ve seen some cases of resistance in patients who didn’t follow their treatment well and therefore relapsed”.*

(Nurse, Côte d’Ivoire)

*“When the physician prescribes a drug to a patient, he or she doesn’t take the time to explain how the patient is going to take it (adherence), hence the problem of resistance”.*

(Pharmacist, DRC)

*“Resistance only occurs when treatment is given poorly. If treatment is given well, there is no resistance”.*

(Nurse, DRC)

*“The resurgence of resistance to malaria treatment is there. This is due to the lack of compliance with treatment in general. But personally, I haven’t seen anyone who has developed resistance”.*

(Nurse, Senegal)

*“The molecule may not have any problem, but the administration may be bad. Or the administration is good, and the medicine is bad because it is counterfeit”.*

(GP, Cameroon)

### 3.2. Challenges to the Implementation of MFT

Survey participants were asked to identify potential challenges to the implementation of an MFT strategy, regardless of the type of approach that would be adopted. The first barrier identified by central-level respondents was the lengthy process of revising malaria policies and guidelines. Although such revisions are carried out on a regular basis, it is time consuming and can delay the uptake of new interventions, especially in countries that recently revised their guidelines. In addition, survey participants indicated that whatever the strategy, it needs to be endorsed and implemented at the global level to effectively address the emergence of resistance. This raises a regulatory challenge, as all countries will have to include the new strategy in their guidelines. Failure to do so could result in some cross-border or parallel import, and will limit the impact on the emergence of resistance.

Funding was another important area of concern. Central-level participants commented that current funding is not sufficient, both from a domestic and international point of view, which will make it difficult to scale up a new intervention. In the case of international funding, some participants indicated that it is fragmented as they rely on different funding sources to cover their whole country. To be able to implement a new intervention at national level, all donors need to coordinate and agree on the approach. Central-level participants included the expected extra cost associated with training and education of healthcare staff and patients in the funding issue. They were concerned by potential confusion among healthcare workers and patients when introducing a new intervention. A robust communication and training plan will need to be put in place to avoid this issue.

Lastly, central-level participants from Nigeria and DRC mentioned the necessity to engage with the private sector. They believed that if the private sector is properly regulated by governments, and the intervention is centrally coordinated, the private sector could be a good partner in the implementation of an MFT strategy. To ensure the support of the private sector, there should be sensitization of the sector on key sections of the drug policies and treatment guidelines; provision of incentives to stock registered ACTs; and training of healthcare workers based in private facilities.

End-user participants were asked to estimate how long it will take to adopt a MFT strategy, regardless of the approach, and to identify any potential bottlenecks. In terms of timing, participants believed that adoption of an MFT strategy should take 3 to 6 months, although answers varied greatly from a few months to up to two years. The reason for this gap is linked to the preparedness of the health facilities, with the most remote ones expected to have more issues with stock management, drug supply, and acceptance by healthcare workers and patients.

*“Once the policy is adopted, I think the longest thing will be orientation and training of health workers”.*

(Nurse, Uganda)

Other end-user participants indicated that because the structure of the healthcare system is pyramidal, it is necessary to convince every level of the pyramid, and moving from one level to the next one takes time (Mali, Senegal). The first step is to convince the authorities so that they agree to adapt national policy guidelines, and then disseminate the document. Then, authorities need to present solid arguments to hospital managers to obtain their endorsement. The next step consists of getting support from the health workers themselves, which should largely be achieved through training. Finally, the information needs to reach the population and requires robust education programs to make sure that the new intervention is endorsed.

*“The decisions come to us from the national level, the regional level, and the district level, so that for each unit, for each area, there are protocols that are agreed upon”.*

(Pharmacist, Mali)

*“Getting every health care professional to understand that this is the transition from old to new is really going to be a challenge, but making it work on the regulatory work is as challenging”.*

(GP, Nigeria)

*“Training health workers and educating patients. For every policy we’ve tried to implement, that’s been the challenge”.*

(Pharmacist, Nigeria)

### 3.3. Perception of the Segmentation Approach

Feedback from central-level participants on the segmentation approach was that it is aligned with the existing strategy of population stratification for the clinical management of malaria (Nigeria, DRC). The benefits of adopting a segmentation approach based on demographic groups enables the monitoring of efficacy and ensures good practice (DRC). In addition, central-level participants expect this approach to be easy to adopt and implement from a technical and logistical perspective because it will leverage the existing supply chain mechanisms.

*“What may be easier to apply in the field is the segmentation approach. It’s going to be less complicated since all the antimalarials will be in the field”.*

(GP, Côte d’Ivoire)

Some end users believed that they were already using a segmentation approach as patients are treated differently according to their specificities. For example, pregnant women do not receive AL during the first trimester of their pregnancy, and they are also not treated with SP as this molecule is used for prevention (Senegal).

Central-level participants expressed a number of potential challenges to the segmentation approach. First, they were concerned by the insufficient supply of ACTs for each population segment. They feared that the shortage of one drug would mean that healthcare providers would use another drug that is not intended for that population. This fear was also mentioned by some end users who felt that the segmentation approach would limit their flexibility in case they experienced stock-outs of one molecule but not the other.

*“As there is that segmentation of different age ranges, it means that all the time there has to be a hundred percent the availability of medicines for different age ranges, and when one age range runs out of medicines, that age range will face problems of the lack of medicines-according to the approach”.*

(Development partner, DRC)

Some central-level participants also emphasized the difficulties of supplying rural areas and areas of conflicts. This supply issue is not specific to the segmentation approach or malaria treatment, but it could potentially derail the segmentation approach as healthcare providers will have to provide whatever treatment is available in these hard-to-reach areas, even if it is not the one intended to this age group.

Another area of concern for central-level participants was the implementation of the segmentation approach in the private sector. Participants stressed that the segmentation approach needs to be implemented in both the public and the private sector to successfully address the resistance issue. If the private sector sources and delivers drugs that are not part of the MFT strategy, it would compromise the whole intervention (Nigeria). In addition, some end users indicated that patients who are not satisfied by the treatment delivered in the public sector source their treatment from the private sector. If the private sector is not fully on board with the MFT approach, it is very likely that patients will receive a different treatment than what is required for the segmentation approach. On a similar topic, self-medication was also mentioned as a limitation to the segmentation approach (Côte d’Ivoire, Uganda).

Lastly, central-level participants expected the segmentation approach to trigger high expenditures on the training of healthcare workers for the correct prescription of ACTs, in addition to a regular education campaign for the local population. The need for training was also stressed by end users who expected that significant education of both healthcare providers and the general population will be required to explain why they need to take one drug and not another, and why different people are using different drugs.

Opinions were mixed regarding the ability of the segmentation approach to address resistance to ACTs. Some end users argued that each segment of the population will actually receive the same drug all the time, hence a given segment of the population is constantly exposed to the same molecule. Others believed that the real cause of resistance is due to human behavior in the form of lack of awareness and self-medication. They claimed that changing the molecules will not address this issue.

*“Prescribing a single combination for an age group is like prescribing a single long-term molecule to that age group”.*

(Nurse, Senegal)

*“The problem is not the segmentation. The problem is how to be sure that each group accesses what you want them to access”.*

(GP, Nigeria)

On the other hand, some respondents felt that the segmentation approach was a good way to fight resistance as the choice of treatment molecule will change during the patient’s life.

*“It can be a good approach because [the molecule changes] when the patient goes from one stage to another. For example in children we use this molecule, when the child become adolescent and adult, he takes a different product than when he was a child. Changing the product will mean it is more effective and can decrease the resistance”.*

(MD, Senegal).

### 3.4. Perception of the Rotation Approach

Very few benefits were mentioned by central-level participants regarding the rotation approach. One participant in Uganda believed it could alleviate the perception of regional discrimination as everyone receives the same drug. A development partner from Uganda suggested that the rotation approach could be an opportunity for older drugs such as chloroquine to be used again. From a resistance point of view, the only benefit of the rotation approach was that it would allow the re-use of products after a period of time.

*“If the efficacy of the candidate ACT begins to decrease, you remove it from the policy and within five years or ten years you can reintroduce it”.*

(NMCP, DRC)

*“The rotation approach is better because we are not going to use a single molecule/combination for a long time and therefore no resistance”.*

(Nurse, Senegal)

From an end-user perspective, the main benefit of the rotation approach is the fact that all patients receive the same product, which limits the chances of making prescription errors.

*“I don’t think there will be room for errors since it is for all age groups”.*

(Nurse, Uganda)

It also facilitates patients’ acceptance as everyone is receiving the same drug. This is especially useful when patients think some drugs are more efficacious or safer than others. On the other hand, end users feared that if patients are not convinced by the efficacy of the drug used, they will look for their preferred drug choice in the parallel market, and the purpose of the rotation approach will be defeated.

The frequency of rotation was at the heart of the discussions with central-level participants, with the optimum interval ranging from two to five years. The reasons driving this interval were the necessity to adapt and optimize the healthcare system and allowing for enough data to be collected on the effectiveness of the intervention (Cameroon, DRC, Nigeria). Activities that need to be put in place include the sensitization of healthcare providers and the public, healthcare workers’ capacity building, restructuring of the supply chain, and coordination of procurement and distribution networks. In addition, robust surveillance systems need to be set up to ensure that drugs with waning efficacy are immediately identified and replaced.

End users on the other hand indicated that the rotation frequency should be every three to six months, and not higher than 12 months. This was driven by the perception that the parasite mutates rapidly, and a short rotation period is necessary to avoid the emergence of resistance. The caveat with a short rotation period is that it will require healthcare staff to be constantly trained, and there were some concerns around acceptance from the patients to always changing the drug.

*“I wouldn’t want to frequently change something that works for my community. If this particular molecule works for them, they may continue to use it beyond a year”.*

(CHW, Nigeria)

In the SMC-implementing countries, end users suggested rotating the drugs at each rainy season.

*“It is true that we have malaria cases all year round, but we have more malaria cases in August to October. I propose to make a first medication [available] during this period, then spread it throughout the year. Then wait for a new peak next August to take a new molecule”.*

(Pharmacist, Senegal)

*“The rotation system has to be based on a specific parameter: the climate. During the winter period, there is an upsurge of mosquitoes […] the rotational use will have to be made in relation to that”.*

(GP, Mali)

Another argument expressed by end users in favor of a short frequency of rotation was for patients who contract malaria more than once per year. With a rotation from 6 to 8 months, they would get a different drug each time.

*“An observant person can have a malaria treatment once a year or even twice at the most. If a patient has malaria in January and has a check-up 6 months later and it is positive, he can enter the new rotating wave”.*

(Pharmacist, Côte d’Ivoire)

Among the challenges identified by central-level participants for the implementation of the rotation approach, some were specific to the intervention while others were difficulties inherent to healthcare management in general. The general challenges included the shortage of staff, regular stock-out, and the weakness of the drug delivery system, especially in large countries like DRC. The challenges relevant to the rotation approach included the different dosing schedules, side effects, and tolerability of the drug used. They feared that patients and providers will have strong views on which drug they prefer, and it could complicate the implementation of the intervention. They were also concerned by a potentially significant wastage of the product when it is time to switch to a different molecule. Another worry concerned the regulatory requirements of such an approach. Guidelines would need to be adapted each time they switched drug, which can be a lengthy process. Lastly, they were concerned about how to cascade the information down efficiently and quickly to the end users.

*“If it is necessary to make rotations, it includes that each time such a policy is changed, it is necessary to follow up on the change with a lot of resources for the population and healthcare providers to be able to effectively use the approach already put in place. The government doesn’t even have the money to pay for these medications, so, to rotate or change policies comes with a number of drawbacks”.*

(Academic researcher, Cameroon)

From an end-user point of view, there were concerns around increase in self-medication and reliance on the black market to obtain the drug that patients want. Another concern was around the need to constantly train the staff and educate the population to avoid confusion. This concern was expressed in a context of a rotation frequency of less than one year.

*“I think it’s going to be confusing when you change too much like that”.*

(Nurce, DRC)

*“Even once health workers are ready to adapt to it, it will create a lot of confusion for people”.*

(Nurse, Nigeria)

End users also identified the logistics constraints of drug rotation as a potential issue. They feared that stock management would not be up to speed and that more than one drug will be available and used at the same time.

*“What is feared is the supply or the fact that the two molecules are found at the same time at the level of the structures”.*

(GP, Senegal)

*“I think it’s a better way to fight resistance, but there should be a single molecule present everywhere during the rotation period”.*

(GP, Côte d’Ivoire)

A few end-user participants also expressed concerns that the rotation approach will lock them in using a product that is not working anymore, or that they would not be able to offer another option for a patient whose treatment failed.

*“The rotation approach will have related technical and management issues because if you are restricted to the rotating drug and you get a catastrophe like resistance, it will be very difficult to wait until next year to treat those cases”.*

(GP, Uganda)

*“You don’t want to tie your neck by giving a product that won’t work for 12 months”.*

(GP, Cameroon)

### 3.5. Perception of the Geographic Approach

According to central-level participants, the main benefit of the geographic approach is the ability to closely monitor drug supplies within a specific region, with participants from Cameroon stating that this approach is already in place in their country.

*“I normally believe in the geographic deployment that Cameroon has adopted where there are efficacious medications in different regions”.*

(Academic researcher, Cameroon)

In order to successfully address the resistance issue, central-level participants indicated that it would be necessary to conduct local therapeutic efficacy surveys prior to deciding which drug to distribute in which region. This is seen as necessary to ensure that there are no existing resistance profiles. Participants warned that this process could take some time and could delay the implementation of the intervention.

Some central-level participants were concerned that the geographic approach might exacerbate perceived regional preferences. This fear was also mentioned by some end users from Nigeria, Uganda, and DRC. According to them, having different drugs in different regions could be negatively perceived by the populations who may think that some areas are more privileged than others.

*“People may say “Don’t take this antimalarial they are giving you because they are giving something different in the Southwest, it is Tinubu (the Lagos sponsor) who is sponsoring this stuff, they use it to kill us”.*

(GP, Nigeria)

*“I think it will be difficult to convince the public of the reasons for using this in the North and that in the East. Is that segregation or what? Some drugs are more expensive than others and the public may see this as segregation”.*

(Pharmacist, Uganda)

The healthcare staff’s high turnover and population mobility were also mentioned as barriers to the implementation of this strategy by both central-level participants and end users: healthcare professionals who move to a different area need to be trained on which drug to use in this specific region. In their view, the fact that the population are very mobile will make this approach impossible to apply systematically (Côte d’Ivoire, DRC), and will also limit its potential to slow the spread of resistance (Nigeria).

*“This geographic approach should not be applicable to Mali given the fluidity of the Malian population”.*

(GP, Mali)

A majority of end users struggled to see the benefits of the geographic approach. For end-user respondents based in SMC countries, this geographic approach created more confusion as they were unsure how it would work with SPAQ (the drug used for SMC). One central-level respondent from Cameroon expressed similar concerns. Most end-user participants argued that because the *P. falciparum* parasite is the same throughout the country, there is no rationale for using different drugs in different regions (Côte d’Ivoire, DRC, Mali, Senegal).

*“We need to do an epidemiological study to see if the same vector is not found everywhere. The geographical approach should be based on the prevalence of this type of plasmodium from one region to another”.*

(Pharmacist, Senegal)

*“As a prescriber, I’m still against it unless the studies tell us that the plasmodium from there are not the same here. But if it is the same, you have to give the same ACTs”.*

(GP, Mali)

Lastly, some central-level participants expressed concerns around funding of the intervention. Some participants indicated that the geographic approach would have to be acknowledged and subscribed to by all funders of the malaria program in order to make sure that all countries follow the same approach. Others warned that different ACTs have different costs, and they feared that more stock-out could occur in regions where the most expensive drugs are allocated compared to regions that use cheaper products. Concerns around fund allocation and differences in ACT costs were also expressed by some end users.

## 4. Discussion

The purpose of this study was to understand the perspectives of decision makers and end users regarding the implementation of MFT strategies as a tool to mitigate the emergence and spread of resistance to ACTs. A qualitative study was conducted in seven Sub-Sahara African countries, involving in-depth individual interviews with key actor groups at various levels of the malaria management landscape. Several key enabling mechanisms for deploying MFT as well as a number of barriers emerged from the data. The survey revealed that convincing evidence of malaria resistance in participating countries was lacking at the time of the study. Survey participants thought it was primarily due to the absence of a robust surveillance system capable of identifying the true causes of treatment failures and monitoring the emergence of resistance to treatment. Specifically, our analysis indicated that, in the absence of such tools, end users attributed treatment failures to a broad range of causes, including self-medication, incorrect dosage, and poor compliance. This issue was further underscored in a recent publication by Takyi et al. (2023) [22], estimating that one in four people with malaria was at risk of receiving sub-optimal antimalarial drug dosing, potentially leading to treatment failure and an increased risk of resistance to malaria drugs. Despite the lack of robust evidence on the emergence of resistance to ACTs, our survey found that all participants were concerned about the consequences of such resistance on the management of malaria. Recent publications providing alarming evidence of the emergence of resistance to artemisinin in several SSA countries [23,24,25,26] prompted the WHO to publish a strategy to respond to antimalarial drug resistance in Africa [11]. Establishing a strong routine surveillance system is one of the five transversal enablers identified by the WHO to ensure the successful implementation of their strategy to respond to antimalarial drug resistance. According to the WHO (2022) [11], a strong routine surveillance and response system would allow “monitoring the local specificities of each setting and further developing locally driven research”. This implies that enough funding and capacity building are allocated to the strengthening of malaria surveillance systems. It also requires the development of tools and communication campaigns to ensure the correct administration and compliance with treatments.

As MFT has been suggested as a strategy to deliver ACT diversification and potentially expand the lifespan of existing ACTs [11], our study sought to identify potential challenges and bottlenecks that the implementation and scale-up of such an intervention could face. From a policy perspective, survey participants indicated that the new MFT intervention would require endorsement at the national, regional, and global levels to ensure its inclusion in guidelines, which is a prerequisite for adoption. The concern is that the process of updating the guidelines can be very lengthy and could lead to a delay in the adoption of the intervention [27,28]. At the national and sub-national levels, end users indicated that the pyramidal structure of the healthcare system is likely to slow down the adoption process of new healthcare interventions. This slowness is problematic as urgent actions need to be taken to fight the emergence of resistance to malaria drugs. Funding was another major bottleneck identified during our analysis. Survey participants were concerned about potential extra costs that the adoption of a new MFT intervention would generate, as they expected that additional training of healthcare workers would be needed, as well as the adaptation of drug delivery mechanisms, and higher costs of some drugs. These concerns were expressed in the context of scarce resources at both the national and international levels, in addition to responding to other issues such as the COVID-19 pandemic. Lastly, end users were concerned about issues linked to health center preparedness, including stock management and drug supply difficulties, as well as acceptance by healthcare staff and patients. The potential challenges identified in our study on the regulatory, funding, resources, education, and supply chain components of a new intervention are consistent with those previously reported [13,29,30]. Notably, the availability of financial, human, and material resources have been identified as the most prominent factors influencing the scale-up of public health interventions, while advocacy and collaboration such as community engagement and partnerships could facilitate adoption and scale-up [29]. This is also in line with the WHO’s strategy to respond to antimalarial drug resistance in Africa in which country ownership, regional coordination, financing, and advocacy efforts have been identified as key enabling mechanisms to ensure the feasible, impactful, and sustainable implementation of interventions such as MFT [11].

The private sector is a major provider of malaria treatment in many African countries and is estimated to sell or dispense between 49% and 92% of antimalarials [31,32,33]. For that reason, failure to integrate the private sector into any MFT strategy was identified as a potential weakness by survey participants, especially those from Nigeria and DRC where the private sector represents a large share of malaria treatment [34]. In the absence of endorsement from the private sector, patients are likely to receive a different treatment than the one recommended in the guidelines. To mitigate that risk, our analysis showed that it will be necessary to sensitize the private sector to the issues caused by resistance to antimalarials, provide financial support to ensure that they are using the registered ACTs, and ensure the training of healthcare workers based in private facilities. The findings are consistent with a recent study investigating African country readiness for the adoption of triple ACTs (TACTs), another strategy envisaged for the mitigation of antimalarial drug resistance [35]. Bridging the gap between the public and the private sectors could build on the experience of the Affordable Medicines Facility for malaria (AMFm) program, which was an experimental financing mechanism designed in 2010 by The Global Fund to increase the availability, affordability, and use of ACTs in the private sector [36]. Although findings indicated that the AMFm program contributed to making ACTs more available in the private sector and remote areas, the program was not scaled up following the pilot period due to funding and sustainability issues [37,38,39]. From an ACT resistance mitigation perspective, the WHO recommends engaging with the private sector by regularly assessing adherence to national treatment guidelines and working through private sector distribution channels to ensure the availability of high-quality ACTs [11]. These recommendations will need to be tailored to the specific context of each country, depending on the existing level of collaboration between the private sector and the government.

Concerning the MFT deployment strategies, our study revealed that each approach had specific advantages and disadvantages, and that participants had a slight preference for the segmentation strategy due to their perception that this approach is already in place in their country. This was largely driven by the treatment of pregnant women who do not receive AL in the first trimester of pregnancy, and who are also not treated with SP as it is already used in prevention. Another benefit of the segmentation approach is that its deployment requires minimal adaptation as it would leverage the existing supply chain mechanisms. These elements generated a sense of familiarity with the segmentation approach, which led to a slight preference for this deployment strategy over the other options. Similar findings were reported by Kabore et al. (2023) [17] who showed that implementing MFT with a segmentation approach was operationally feasible and acceptable in Burkina Faso. In our survey, the main concern associated with the segmentation approach was a shortage of drugs. Survey participants feared that an out-of-stock situation of one drug would derail the whole approach, as patients will need to receive drugs that are intended for other groups. The fact that various ACTs have different costs could exacerbate the supply issue. The rotation approach could mitigate this problem as all patients receive the same drug at the same time, which facilitates drug supply. In addition to stocking benefits, the rotation approach limits the risk of prescription errors, as all patients receive the same treatment. Consequently, survey participants believed that less training of healthcare staff would be required as they only need to know how to use one drug. Lastly, the rotation approach facilitates patients’ acceptance as they all receive the same drug. The main weakness of the rotation approach is finding the optimal frequency of rotation. Views varied greatly between national stakeholders who envisaged a frequency of rotation ranging from two to five years, while end users recommended a rotation of 12 months or less. These discrepancies were due to differences in focus from the two groups: end users believe that a short rotation frequency is needed to limit the emergence of resistance, while national stakeholders prefer a long rotation frequency for logistic, cost, and training reasons. Further work is required to identify how frequently to rotate ACTs to slow down the emergence of resistance, and to better understand the tradeoff between the efficacy of the intervention versus the logistic hurdles and costs associated with the rotation of antimalarials. Finally, the geographic approach was the least desirable deployment strategy, as it could exacerbate regional differences. From an implementation point of view, the geographic approach requires conducting therapeutic efficacy surveys (TES) to identify which drug to use where. Conducting such TES is costly and may take too much time before the results are available. No significant benefit of the geographic approach was identified by our survey.

### Limitations

The findings reported in this study present a number of limitations. The first limitation lies in the generalizability of the findings to all malaria-endemic countries. Many important local characteristics such as changes in malaria incidence and prevalence, the speed of resistance emergence to ACTs, and the coverage of interventions such as seasonal malaria chemoprevention or mass drug administration could significantly impact the uptake of MFT and will vary per country. To address this limitation, the study included seven countries possessing distinct profiles in terms of malaria burden and potential emergence of resistance to ACTs. Another limitation stems from the relatively small number of respondents per type at country level. Despite this constraint, the study maintained a sufficient number of participants at the aggregate level, ensuring the robustness of the overall findings. A third limitation pertains to the absence of patients in the sample. This exclusion was based on the rationale that patients’ perceptions are better captured through feasibility studies which was not the focus of this study. Authors should discuss the results and how they can be interpreted from the perspective of previous studies and of the working hypotheses. The findings and their implications should be discussed in the broadest context possible. Future research directions may also be highlighted.

## 5. Conclusions

This qualitative study identified several potential barriers and enablers of deployment of MFT strategies to mitigate the emerging resistance to ACTs in Africa. The findings underscore the critical need for a strong routine surveillance system to monitor the emergence of resistance to ACTs. This was driven by the apprehension about the consequences that the emergence of resistance to ACTs would have on malaria treatment. The study also highlighted the necessity for a national, regional, and global endorsement of MFT deployment strategies and called for a collaborative approach through the engagement of stakeholders at all levels of malaria management. In particular, the introduction of MFT will require alignment with the financial, policy, and advocacy components of malaria care. In addition, the integration of the private sector into MFT strategies was underscored as crucial to ensure widespread and coordinated adoption of MFT. The study insights contribute to the ongoing dialogue on effective malaria drug resistance intervention strategies, emphasizing the need for a multifaceted and context-specific approach.

## Figures and Tables

**Table 1:** Characteristics of selected countries.

Country	High Burden Country	ACTs Registered for First-Line Malaria Treatment
Cameroon	Yes	AL, ASAQ, DHA-PQ, ASPYR
DRC	Yes	AL, ASAQ
Ivory Coast	No	AL, ASAQ, DHA-PQ, ASPYR
Mali	Yes	AL, ASAQ, DHA-PQ, ASPYR
Nigeria	Yes	AL, ASAQ
Senegal	No	AL, ASAQ
Uganda	Yes	AL, ASAQ

DRC: Democratic Republic of Congo, ACTs: artemisinin based treatments, AL: artemether lumefantrine, ASAQ: artesunate amodiaquine, DHA-PQ: dihydroartemisinin piperaquine, ASPYR: pyronaridine artesunate.

**Table 2:** Sample composition per country.

	Cameroon	DRC	Ivory Coast	Mali	Nigeria	Senegal	Uganda	Total
Central level:								
NMCP/MoH	0	2	1	1	5	2	0	11
Academia/research	1	1	1	2	2	1	1	8
Development partners	3	3	2	1	3	1	1	13
End users:								
Physicians	7	7	7	12	7	7	7	54
Pharmacists	7	7	7	7	7	7	7	49
Nurses	5	8	5	5	6	5	5	39
CHWs	5	2	5	0	4	5	5	26
Total	28	30	28	28	34	28	26	200

NMCP: National Malaria Control Program; MoH: Ministry of Health; DRC: Democratic Republic of Congo.

## Data Availability

The dataset supporting the conclusions of this article is included within the article (and its Supplementary Materials).

## Supplementary Materials

The following supporting information can be downloaded at: https://www.mdpi.com/article/10.3390/tropicalmed9050093/s1, File S1: Discussion guides.
