# Supplementary material for: Evaluation of Segmentation, Rotation, and Geographic Delivery Approaches for Deployment of Multiple First-Line Treatment (MFT) to Respond to Antimalarial Drug Resistance in Africa: A Qualitative Study in Seven Sub-Sahara Countries
Source: Trop Med Infect Dis. 2024 Apr 23;9(5):93. doi: 10.3390/tropicalmed9050093 (PMC11125622; doi:10.3390/tropicalmed9050093)
Supplement: Supplementary file 1 [file tropicalmed-09-00093-s001.zip › tropicalmed-2946451-supplementary.pdf]

End-users discussion guide

1. There are reports that antimalarial resistance is on the increase and a danger to the malaria control efforts.
    a. What is your opinion on this in your environment? Have you, or someone close to you, had experiences with resistance? If so, how does it impact your desire to find solutions?
    b. What do you know about the issue of resistance to artemisinin? In general, and your environment?
    c. How acute do you think this resistance is? Out of 10 patients you see, how many would you say experience resistance to artemisinin?
    d. Do you think the current efforts to mitigate the problem are sufficient? Why do you say so?
2. On multiple first-line therapies (MFTs) as a strategy to delay the emergence of malaria parasite resistance:
    a. Could you share with me what you know on MFTs?
    b. Where did you learn what you know from?
    c. Do you know anyone else in your environment who knows about MFTs? If so, how would you compare their knowledge of MFTs with yours?
    d. How would you characterize the discourse on MFT in your environment with regards to policy, perceptions on usefulness and implementation strategies?
    e. Based on what you know, describe how malaria control in the health sector is currently prioritized and the appetite for a new treatment approach like MFT?
    f. In your opinion, where are the major bottlenecks/challenges to a possible introduction and roll-out of MFT?

Deploying multiple first-line combination therapies allows to challenge parasite populations with many different types of drugs, and thus delay and slow down drug resistance evolution more than with a single combination therapy. A modelling study demonstrated that MFT was predicted to reduce the long-term number of treatment failures compared with strategies in which a single first-line ACT is recommended. Inclusion of a single non-ACT therapy in an MFT strategy would have substantial benefits in reduction of pressure on artemisinin resistance evolution, delaying its emergence and slowing its spread.

Several MFTs implementation scenarios can be imagined, including distribution of one ACT for home-based care and a different one for clinic use, partition of the ACTs market by segments of the population (pediatric patients, pregnant women, adults), alternating distribution of different ACTs over a given period of time, and distribution of different drugs in different geographical regions.

3. What are your thoughts about these different implementation strategies for MFTs:

    A trial in the district of Kaya, Burkina Faso, follows the partition approach and enrolled its first patients in December 2019. The trial is designed as follows: patients seeking care at community level receive AL independently from their age, sex or pregnancy, patients seeking care at health facility level receive artesunate-pyronaridine (Pyramax) if they are under 5 years of age while pregnant women receive AL and all other patients get dihydroartemisin-piperaquine (DHA-PQ).
    [Note to the interviewer: There are conflicting messages coming from WHO about the use of Pyramax. It has been approved and prequalified, but not included in the guidelines yet. There is a fight at WHO between supporters of Pyramax and others who think the safety has not been investigated enough. As a result, in some countries (e.g. Côte D'Ivoire) the local WHO representatives are recommending against Pyramax. ***If such concerns are raised by the respondent, pelase ask them to assume that it is fully endorsed by WHO****]*

a. What do you think about this **partition approach**, that is having a specific drug combination for a targeted population (children under the age of 5, pregnant women, and adults)?
   i. If the partition approach was to be adopted in your environment, what would be the target groups in the population?
   ii. Can you comment on the logistics that would be required to implement this approach?
   iii. What challenges do you envisage this approach will face?

      Probe on:
      - [For Pharmacist only] Procurement (managing synchronized procurement cycles)
      - [For Pharmacist only] Logistic challenges, (supply, safety stock, alignment with shelf life)
      - Training of staff delivering the treatment
      - Acceptance of patients
      - Confusion/difficulty in adapting to the new strategy among users
      - Educational messages needed for the patients

Kenya is setting up an alternating distribution trial in which one county will be offered an 8-month rotational use of AL, AS-AQ and DHA-PQ (8 months of AL, followed by 8 months of AS-AQ, followed by 8 months of DHA-PQ), a second county will have a 12-month rotational use of AL and Pyramax (12 months of AL followed by 12 months of Pyramax), and a third county will serve as control by using AL during the total duration of the trial (24 months). *[Note to the interviewer: There are conflicting messages coming from WHO about the use of Pyramax. It has been approved and prequalified, but not included in the guidelines yet. There is a fight at WHO between supporters of Pyramax and others who think the safety has not been investigated enough. As a result, in some countries (e.g. Côte D'Ivoire) the local WHO representatives are recommending against Pyramax.* ***If such concerns are raised by the respondent, pelase ask them to assume that it is fully endorsed by WHO****]*

b. What do you think about a **rotational** use of ACTs?
   i. If rotational use of ACTs was to be adopted in your environment, what would be the ideal frequency of the rotation?
   ii. Why do you say that frequency is ideal and what informs your thinking?
   iii. Can you comment on the logistics that will be required to implement this strategy?
   iv. What challenges do you envisage this approach will face?

      Probe on:
      - [For Pharmacist only] Procurement (managing synchronized procurement cycles)
      - [For Pharmacist only] Logistic challenges, (supply, safety stock, alignment with shelf life)
      - Training of staff delivering the treatment
      - Acceptance of patients
      - Habits of HCPs and patients as hindrances?
      - Educational messages needed for the patients

   v. [For MD only] About you personally, in your daily practice:

- Do you see yourself prescribing different first-line molecules/combination of molecules (i.e. rotate the prescription of molecules/combinations) against malaria? If no, can you please tell me why?
- Is/Are there any molecule(s)/combination(s) of molecules that you are not willing to prescribe? If yes, can you please tell me which one(s)?

c. What about having a **different geographical** use of ACTs?
   i. If the geographical use of ACTs was adopted in your environment, what will inform which region gets which ACTs?
   ii. Comment on how suitable this would be given your knowledge of malaria epidemiology in your environment.
   iii. What challenges do you envisage this approach will face?

   Probe on:

   - [For Pharmacist only] Procurement (managing synchronized procurement cycles)
   - [For Pharmacist only] Logistic challenges, (supply, safety stock, alignment with shelf life)
   - Training of staff delivering the treatment
   - Acceptance of patients
   - Habits of HCPs and patients as hindrances?
   - Educational messages needed for the patients

d. Based on your views and understanding on the different approaches above, what would you say is the best approach for an MFTs strategy in your environment? Why do you say that?

4. On the uptake of MFT by your health center:
   a. How long do you anticipate it would take your health center?
   b. Which steps will take the longest in your opinion?
   c. What makes you think so?
5. Is there anything else that we have not discussed that you want to share with us? Or anything you would like to add / have forgotten on the topics discussed today?

Central level respondents discussion guide

1. There are reports that antimalarial resistance is on the increase and a danger to the malaria control efforts.
    a. What is your opinion on this in your country?
    b. To what extent are these concerns valid in your context with regards to the size of the problem, nature of evidence, and the current efforts to mitigate the problem?
2. **What do you know about multiple first-line therapies (MFTs) as a strategy to delay the emergence of malaria parasite resistance?**
    a. Where did you learn what you know from?
        i. How knowledgeable would you say you are regarding the MFTs strategy?
    b. How would you characterize the discourse on MFT in your country?
        i. What are the existing perceptions on the usefulness of the strategy?
        ii. What is the policy position?
        iii. What are the implementation strategies?
    c. Based on what you know, observed or heard in the government circles, describe how malaria control in the health sector is currently prioritized and the appetite for a new treatment approach like the MFT.
        i. Can you share any evidence for this opinion in terms of policy development, financing etc.?
    d. In your opinion, where are the major bottlenecks/challenges to a possible introduction and roll-out of MFT in your country.
        i. How can some of these anticipated challenges be addressed?

Several MFTs implementation scenarios can be imagined, including distribution of one ACT for home-based care and a different one for clinic use, partition of the ACTs market by segments of the population (pediatric patients, pregnant women, adults), alternating distribution of different ACTs over a given period of time, and distribution of different drugs in different geographical regions.

3. **What are your thoughts about these different implementation strategies for MFTs?**
    a. What do you think about the **partition approach**, that is having a specific drug combination for a targeted population (children under the age of 5, pregnant women, and adults)?
        i. What benefits will the **partition approach** bring in delaying and slowing down drug resistance.
        ii. If the partition approach was to be adopted in your country, what would be the target groups in the population?
        iii. Can you comment on the logistics that would be required to implement this approach?
        iv. What challenges do you envisage this approach will face?
            Probe on:
            - Funding (pricing and bargaining power disadvantage, higher cost of some ACTs).
            - Procurement (managing synchronized procurement cycles)
            - Logistic challenges, (supply, stock management, safety of stock, alignment with shelf life)
                - E.g. ASK: Do you think inventory taking and stock redistribution can avoid wastage due to slow movement of products or dead stock in some facilities?
            - Training of staff delivering the treatment

- Acceptance of patients,
- Confusion/difficulty in adapting to the new strategy among users

b. What do you think about a **<u>rotational</u>** use of ACTs?
   i. If rotational use of ACTs was to be adopted in your country, what would be the ideal frequency of the rotation?
   ii. Why do you say that frequency is ideal and what informs your thinking?
   iii. Can you comment on the logistics that will be required to implement this strategy?
   iv. What benefits will the rotational use of ACTs bring in delaying and slowing down drug resistance in the country?
   v. What challenges do you envisage this approach will face?
      Probe on:
      - Funding (pricing and bargaining power disadvantage, higher cost of some ACTs).
      - Procurement (managing synchronized procurement cycles)
      - Logistic challenges, (supply, safety stock, alignment with shelf life)
        - E.g. ASK: Do you think inventory taking and stock redistribution can prevent wastage due to slow movement of products or dead stock in some facilities?
      - Training of staff delivering the treatment
      - Acceptance of patients

c. What about having a **<u>different geographical</u>** use of ACTs?
   i. If the geographical use of ACTs was adopted in your country, what will inform which region gets which ACTs?
   ii. Comment on how suitable this would be given your knowledge of malaria epidemiology in your country.
   iii. What benefits does deploying different ACTs to different regions of the country bring in delaying delay and slowing down drug resistance.
   iv. What challenges do you envisage this approach will face?
      Probe on:
      - Funding (pricing and bargaining power disadvantage, higher cost of some ACTs).
      - Procurement (managing synchronized procurement cycles)
      - Logistic challenges, (supply, safety stock, alignment with shelf life)
        - E.g. ASK: Do you think inventory taking and stock redistribution can avoid wastage due to slow movement of products or dead stock in some facilities?
      - Training of staff delivering the treatment
      - Acceptance of patients

d. Based on your views on the different implementation formats above, what would you say is the ideal format for an MFTs strategy in your country?
   i. Why do you say that?

4. Do you see any benefit in including the MFTs strategy in your national malaria policies and treatment guidelines?
   a. Why/Why not?

5. **What will need to happen for you/your country to start implementing an MFTs strategy?**

    Probe on

    - Health system issues (procurement, supply, human resource,)
    - Policy issues
    - Community mobilization/sensitization

6. **Of the three MFT strategies discussed above, which one will take the longest in uptake in your country?**
    a. What are your reasons why this approach will take long when compared to the other strategies?

7. **What role will the regulator e.g. the pharmacy and poisons board play in the implementation of the MFT strategy in your country?**
    a. What role will the private sector play in supporting the role out of MFT? Which members of the private sector are significantly important in the implementation of the MFT strategy?
    b. Who the OTHER key stakeholders in MFT roll-out?
    c. What role would these OTHER stakeholders play in MFT strategy rollout?
